# Less is More: Lower Levels of Task-Induced Hippocampal Activation Predict Better Performance on a Separate Verbal Memory Evaluation

**DOI:** 10.1101/2025.06.02.656665

**Published:** 2025-06-03

**Authors:** Caitlin A. Sisk, Kathleen B. McDermott, Jed T. Elison, Meghan A. Swanson, Gagan S. Wig, Adrian W. Gilmore, Steven M. Nelson

**Affiliations:** 1 Department of Pediatrics, University of Minnesota, Minneapolis, MN 55454; 2 Masonic Institute for the Developing Brain, University of Minnesota, Minneapolis, MN 55414; 3 Mallinckrodt Institute of Radiology, Washington University School of Medicine, St. Louis, MO 63110; 4 Department of Psychological and Brain Sciences, Washington University, St. Louis, MO 63130; 5 Institute of Child Development, University of Minnesota, Minneapolis, MN 55455; 6 Center for Vital Longevity, School of Behavioral and Brain Sciences, University of Texas at Dallas, Dallas, TX 75235; 7 Department of Psychiatry, University of Texas Southwestern Medical Center, Dallas, TX 75390; 8 Department of Psychological and Brain Sciences, University of Delaware, Newark, DE 19716

**Keywords:** hippocampus, learning, memory, parietal memory network, replication

## Abstract

Memories that differ in content or duration differ in the extent to which they depend on the hippocampus, and also the part of the hippocampus, posterior (pHPC) or anterior (aHPC), that they implicate. Inter-individual differences in learning-related activation in different hippocampal subregions have been found to predict specific differences in memory abilities. The complexity of these relationships creates a setting that is ripe for theoretically informative investigation, but that can also lead to reports of spurious relationships that do not reflect underlying neurobiological associations. Across-study replication is therefore a critical first step toward understanding how differences in hippocampal activity drive individual differences in memory ability. In the domain of verbal memory, Wig et al. (2008) identified a negative relationship between task-induced activation in pHPC and out-of-scanner verbal memory test performance. Replicating this result in an independent sample of 86 participants, we identified the same negative correlation between pHPC activation during a Lithuanian word learning task and out-of-scanner California Verbal Learning Test (CVLT-II) performance. This replication represents a critical step toward understanding how pHPC supports verbal memory by answering the basic question of whether the relationship can be reliably observed.

## Introduction

The hippocampus, seated in the medial temporal lobe, plays a key role in human and non-human animal memory function (Squire, 1992). Although this basic observation has long been appreciated (e.g., (Scoville & Milner, 1957), functional dissociations between anterior and posterior aspects of the structure only became major targets of research sometime later (see Poppenk et al., 2013 for review). Thus, while “the” role of the hippocampus in memory has been the study of psychologists and neuroscientists for many decades, investigations into the nature of how different parts of the hippocampus might differentially contribute to memory or other cognitive processes remain ongoing. A consensus seems to be emerging that anterior aspects of the hippocampus tend to process more abstract information, whereas posterior aspects tend to process fine-grained details (see Maurer & Nadel, 2021).

Given its association with fine-grained information, a natural prediction may be that greater activity during task-based periods, compared to periods of rest, might predict a stronger general memory ability. However, contrary to this prediction, evidence suggests that less activity in the posterior hippocampus during active encoding conditions may instead be a better index of memory performance. Across two studies, Wig et al. (2008) identified, and subsequently replicated, a negative correlation across subjects between task-induced posterior hippocampal activation and performance on out-of-scanner testing of verbal memory. Neither task required explicit encoding demands, and they involved odd-even number judgements or responding to onsets and offsets of a flickering checkerboard. The authors suggested that, as their analyses compared task activation to a resting-state baseline, the results likely indicated consolidation-related mechanisms during rest explained the pattern of results.

The hypothesis forwarded by Wig et al. (2008) would suggest that similar negative correlations should be observed between task-states and out-of-scanner memory performance in different task conditions. In this report, we re-analyze data from a large sample of participants (N = 86) in which individual differences in general learning ability were previously reported (Nelson et al., 2016) based on rates of learning Lithuanian-English word pairs. Using these data, we ask if any correlation between task-activation and out-of-scanner learning might be obtained. As the data from Nelson et al. involve explicit encoding, the task conditions differ substantially from those reported by Wig et al. (2008) and therefore constitute a strong test of the general mechanisms hypothesized in that paper. A critical second question concerned the localization of these effects: if any were observed, would they fall specifically in the posterior hippocampus, as in Wig et al., or would they be located across the hippocampus or medial temporal lobe more broadly?

## Methods

### Participants

One hundred participants were recruited via Craigslist (www.craigslist.org) and community-posted flyers in the St. Louis area. Of the 14 excluded participants, 6 were excluded for excessive movement, 6 for failure to comply with task instructions (failure to keep eyes open or failure to respond), 1 for failure to complete the experiment, and 1 for opting out of participation due to illness. This resulted in a final sample of 86 participants (36 female) between the ages of 18 and 31 years (*mean*: 24.82) who had completed between 10 and 22 years of education (*mean*: 15). Inclusion criteria included right-handedness, English as a native language, and normal or corrected-to-normal vision. Exclusion criteria included reported history of neurological or psychiatric illness. All participants were consented according to the guidelines of Washington University’s Human Research Protection Office and were compensated $25 per hour of participation.

### Stimuli

Stimuli consisted of 45 direct Lithuanian–English translations (e.g., AKIS–EYE) of concrete nouns from published norms (Grimaldi et al., 2010) presented in all capital letters with no typographic ligatures or diacritical marks. Stimuli were presented in white 48-point Arial typeface on a black background. Lithuanian words varied in character length from 4 to 9 (*mean*: 5.96), and English words varied in character length from 3 to 8 (*mean*: 4.56). Lithuanian words varied in number of syllables from 2 to 4 (*mean*: 2.4), and English words varied in number of syllables from 1 to 2 (*mean*: 1.22). Participants reported no prior knowledge of the Lithuanian language.

### Procedure

**Figure 1.**
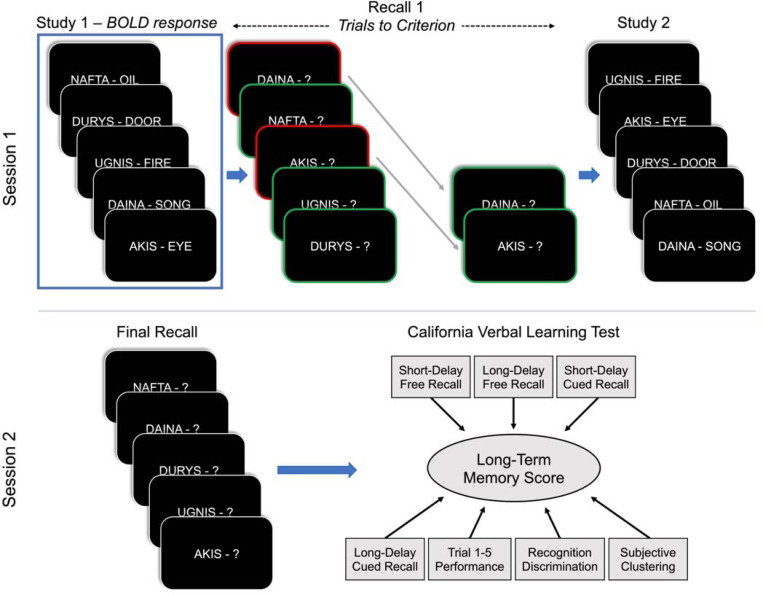
Schematic of the experimental paradigm. In Session 1, participants first studied Lithuanian–English word pairs during Study 1, then they completed iterative cued-recall testing of each word pair until reaching the 100% accuracy criterion during Recall 1, then they re-studied the same word pairs during Study 2. In Session 2, which occurred 2 days later, participants completed a single iteration of the cued-recall test followed by a cognitive battery that included the CVLT-II. BOLD response was collected during Study 1 and during 10-minute resting periods preceding Study 1 and Study 2.

The experimental procedure consisted of four phases across two sessions of a Lithuanian-word-learning task. Phases 1–3 took place during Session 1, and phase 4 took place 2 days later during Session 2 (Fig. 1). The first phase, Study 1, took place inside of the scanner during active collection of blood oxygen level-dependent (BOLD) response. During the Study 1 phase, participants were shown 45 Lithuanian–English word pairs one at a time in random order and were instructed to memorize the word pairs. Participants were told that a later recall test would occur in which they would be shown the Lithuanian cue and would be required to provide the matching English translation. During the Study 1 phase, each Lithuanian–English word pair was presented for 3.5s with jittered interstimulus intervals of 1.5–6.5s. BOLD response was not collected during any other active phase of the experiment.

In the second phase, Recall 1, participants remained inside the scanner to complete iterative cued-recall tests with feedback until reaching the 100% recall criterion. On each cued-recall trial, participants were shown a Lithuanian cue word (e.g., AKIS) for 3.5s and were instructed to verbally recall the matching English translation (e.g., “eye”). Regardless of response accuracy, the correct English word was displayed for 1.5s following each Lithuanian cue presentation. All of the 45-word pairs were presented in random order in the first iteration, and correctly-recalled word pairs were removed from the list of pairs to be tested in future iterations. A 1s interstimulus interval separated each item, and a 30s arithmetic problem solving task separated each iteration of this phase. During the arithmetic task, participants solved arithmetic problems aloud to limit the possibility of working memory maintenance of cue-target pairs from the previous iteration. Participants completed this cued-recall test in repeated iterations, separated by arithmetic blocks, with removal of correctly-recalled word pairs until participants had correctly recalled the English translation of each Lithuanian word cue.

The third phase, Study 2, was identical to Study 1 excepting measurement of BOLD response. Both Study 1 and Study 2 were preceded by 10-minute resting-state scans with participants fixated on a crosshair at the center of the screen.

The fourth and final phase, Final Recall, took place 2 days (*range*: 36.5–49.5h, *mean*: 43h) after the previous phases. During this Final Recall phase, participants were presented with Lithuanian cue words for 8s and were instructed to verbally recall the matching English translation. No feedback was provided. All 45 Lithuanian–English word pairs were tested in a single iteration in random order with 1s interstimulus intervals. Following Final Recall, participants completed a cognitive battery including the CVLT-II memory evaluation.

### fMRI Data Acquisition

A standard set of imaging protocols guided acquisition of functional MR images. Foam pillows and a thermoplastic mask fastened to the head coil stabilized participants’ head position. All images were acquired using a Siemens MAGNETOM Tim Trio 3.0T Scanner (Erlangen, Germany) and a Siemens 12 channel Matrix Head Coil. A T1-weighted sagittal Magnetization-Prepared Rapid Gradient-Echo structural image was obtained (time echo [TE] = 3.08 ms, time repetition [TR] partition = 2.4 s, time to inversion [TI]=1000ms, flip angle=8°, 176 slices with 1×1×1mm voxels) (Mugler & Brookeman, 1990). In addition, a T2-weighted turbo spin echo structural image (TE = 84 ms, TR = 6.8 s, 32 slices with 2 × 1 × 4 mm voxels) was obtained in the same anatomical plane as the subsequent BOLD images to improve alignment to an atlas. An auto-align pulse sequence protocol provided in the Siemens imaging software package was used to align the acquired slices from functional scans in parallel to the anterior commissure-posterior commissure plane and centered on the brain. This plane parallels the slices in the Talairach atlas (Talairach & Tournoux, 1988), which is used for subsequent data analysis. Functional imaging was performed using a BOLD contrast sensitive gradient echo echo-planar sequence (TE = 27 ms, flip angle = 90°, in-plane resolution = 4 × 4 mm). Whole-brain Echo Planar Imaging volumes of 32 interleaved, 4-mm-thick axial slices were obtained every 2.5 s. The first 4 image acquisitions were discarded to allow net magnetization to reach steady state.

Noise-cancelling headphones were used to help dampen scanner noise for participants. The headset was equipped with a microphone and allowed participants to communicate with research technicians throughout the scanning procedures. An Apple iMac computer (Apple, Cupertino, CA, USA) running PsyScope software (Cohen et al., 1993) and Adobe Flash Professional CS5.5 (Adobe Systems, San Jose, CA, USA) was used to display visual stimuli. An LCD projector (model PG-C20XU, Sharp) was used to project stimuli onto an MRI-compatible rear-projection screen (CinePlex) at the head of the scanner bore. Subjects viewed this screen through a mirror mounted to the top of the head coil.

### fMRI Data Preprocessing

Each subject’s fMRI data was preprocessed with a standardized stream meant to reduce noise and remove artifacts from the data. This protocol included: 1) correction for movement within and across runs using a rigid-body rotation and translation algorithm (Snyder, 1996); 2) mode-1000 intensity normalization, allowing for comparisons across subjects (Ojemann et al., 1997); and 3) temporal realignment of all slices to the temporal midpoint of the first slice using sinc interpolation to account for the slice-time acquisition differences. Functional data were then resampled into 3-mm isotropic voxels and transformed to stereotaxic atlas space (Talairach & Tournoux, 1988). To register individuals’ data to an atlas, subjects’ T1-weighted images were aligned to a custom atlas-transformed (Lancaster et al., 1995) target T1-weighted template (711–2B) using a series of affine transforms (Fox et al., 2005; Michelon et al., 2003).

### Administration of Memory Evaluation

The CVLT-II was administered amongst several other cognitive measures. For additional details regarding other measures, see Nelson et al. (2016). The CVLT-II consisted of two parts. In Part 1, the experimenter read a list of 16 words, and the participant was asked immediately after to recall as many of the words as they could remember in any order. This comprised List A Immediate Free Recall Trial 1 through List A Short Delay Cued Recall (Delis et al., 1987). In Part 2, which occurred 20 minutes after Part 1, participants were asked to recall the in any order as many of the 16 words they could remember from the list presented in Part 1. This part included List A Long Delay Free Recall through List A Long Delay Yes/No recognition (Delis et al., 1987). Instructions were read directly from the CVLT-II packet. Participants did not complete the Forced-Choice Recognition portion of the CVLT-II.

### Analysis of Memory Evaluation

Together, Part 1 and Part 2 of the CVLT-II yielded 16 separate memory measures. A factor analysis of CVLT-II memory measures identified seven measures that loaded onto the first component and accounted for 40.82% of the total variance: “short-delay free recall,” “long-delay free recall,” “short-delay cued recall,” “long-delay cued recall,” “trial 1–5 performance,” “subjective clustering,” and “recognition discrimination.” We z-scored these measures and averaged them for each participant to obtain the “long-term memory” score. See Nelson et al. (2016) for a full list of the CVLT-II memory measures and more details regarding the factor analysis.

## Results

**Figure 2.**
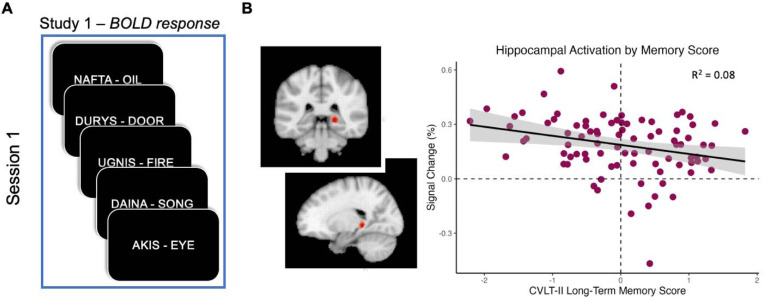
Hippocampal activity correlates with long-term memory performance. Scatterplot depicts the relationship between CVLT-II long-term memory score and signal change in the right hippocampal tail from rest to Study 1 periods.

Participant z-scores on the CVLT-II long-term memory measure ranged from −2.21 to 1.82. There was a significant negative relationship between CVLT-II long-term memory score and level of hippocampal activation relative to rest (*r* = −0.27, *p* = .012), such that participants with lower hippocampal activation relative to rest performed better on the CVLT-II (Fig. 2).

**Figure 3.**
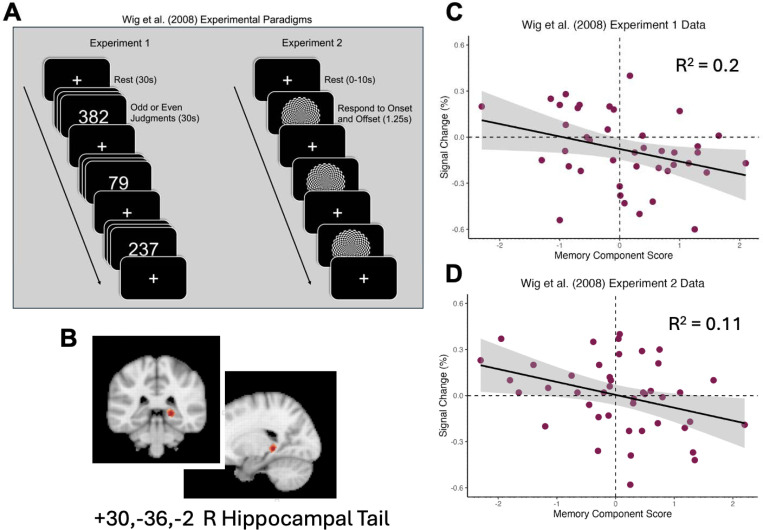
Previously-reported data showing a negative relationship between task-induced hippocampal activation relative to rest and performance on an outside memory battery. (A) Schematics of the experimental paradigms used in Exp. 1 and Exp. 2 of Wig et al. (2008). In Exp. 1, participants alternated between 30s resting state periods in which they fixated on central crosshairs and 30s periods in which they made odd or even number judgments. In Experiment 2, participants indicated the onset and offset of a flickering checkerboard with jittered periods of 0–10s of rest between active trials. (B) Scatterplot of the relationship in Exp. 1 between memory component score and percent signal change in the right hippocampus during the odd or even number judgment task relative to resting periods. (C) Scatterplot of the relationship in Exp. 2 between memory component score and percent signal change in the right hippocampus during the flickering checkerboard task relative to resting periods.

These findings represent a near-direct replication of the findings presented in Wig et al. (2008), in which hippocampal activity at rest, relative to task periods, correlated negatively with a memory component score on a separate memory battery. This was true across two experiments with two different tasks and organization of resting periods (Fig. 3).

## Discussion

Lower levels of pHPC activation during a verbal paired associates learning task predicted better performance on CVLT-II scores obtained outside of the scanner. These results, obtained in an independent sample of 86 young adults, serve as a clear replication of prior results identifying task-evoked pHPC activity as a predictor of individual differences in verbal memory function.

A primary purpose of the present investigation was to provide a critical out-of-sample replication to form a basis for future investigations of individual differences in memory ability relating to hippocampal activation levels. That significant effects were observed in the same region, and in the same direction, as the initial findings of Wig et al. (2008) obviates recently-stated concerns that many individual differences previously observed in smaller-N studies might be spurious (Marek et al., 2022). Indeed, the finding reported here reinforces the need to further explore the mechanisms underlying the relationship between pHPC and verbal memory.

### Hippocampal Markers of Individual Differences in Memory Ability

The present findings complement a large body of previous research demonstrating close relationships between regions of the hippocampus and individual differences in memory performance or capacity. At least three lines of previous research, considered alongside the present findings, support the conclusion that lower task-induced pHPC activation, relative to rest, supports verbal memory ability. First, structure-behavior associations between the hippocampal tail and memory performance highlight pHPC as a region of importance in verbal memory function (Allen et al., 2006; Bettio et al., 2017; Mungas et al., 2002; Poppenk & Moscovitch, 2011). Second, functional links between that region and memory-related brain networks establish pHPC as a key active player in memory-related cognition and task engagement in general (Angeli et al., 2023; Zheng et al., 2021). Third, brain-behavior analyses have in fact previously identified a negative correlation between pHPC activity during an engaging memory task and performance on out-of-scanner verbal memory evaluations (Johnson et al., 2001; Wig et al., 2008). As an extension of many previous investigations and a replication of one, our observation that reduced task-induced pHPC activation differentiated individuals of differing memory capabilities establishes this finding as reliable and sufficiently consistent as to support further investigation.

Recent functional connectivity findings further underscore functional differences between pHPC and aHPC (Angeli et al., 2023; Zheng et al., 2021). In a recent investigation, pHPC, which was previously structurally associated with memory retrieval displayed a functional connection to the parietal memory network (PMN; Gilmore et al., 2015) and salience network (SAL; Seeley, 2019; Seeley et al., 2007), while aHPC was connected instead to the default mode network (DMN; Gusnard et al., 2001; Raichle, 2015; Raichle & Gusnard, 2005). Associations between task-evoked BOLD response and behavioral measures of task performance again differed categorically between the pHPC and aHPC. While aHPC and DMN activity correlated with scene construction in an episodic projection task, pHPC and PMN/SAL activity instead appeared to track target salience or novelty and general task engagement in an oddball detection task (Angeli et al., 2023). These findings mirror a large literature demonstrating connectivity differences between pHPC and aHPC both in humans and in animal models (Barnett et al., 2021; Brunec et al., 2019; Fanselow & Dong, 2010; Poppenk et al., 2013), with recent evidence indicating that the different hippocampal subregions exhibit differing trajectories in their respective connectivity as individuals age.

Differences in function across the hippocampal long axis are important to consider when contextualizing the current findings. The region of the hippocampus identified by Wig et al. (2008) and replicated in the current report lies within pHPC. If, as prior evidence suggests, pHPC activity tracks task engagement or salience (Angeli et al., 2023) and supports the processing of fine-grained details in the context of episodic memory (Poppenk et al., 2013; Sekeres et al., 2018), one might posit that task-induced perturbations are reflective of general mnemonic information processing efficiency. Convergent with this idea, pHPC is strongly functionally connected with both memory- and control-related networks (Zheng et al., 2021). The present findings align with this possibility by replicating prior evidence that posterior hippocampal activity tracks individual differences in a task-general memory measure performed outside of the scanner. A more complete mechanistic explanation of the association awaits future clarification, but the direction and generality of the correlation appears reliable and robust.

### Limitations

Although the present study represents a necessary replication of previously reported findings, there are important limitations to consider. First, the nature of our targeted replication led us to limit analyses of brain-behavior correlations to a region within pHPC. While benefitting from strong theoretical motivation, it is necessarily limited to a small portion of a single structure. A whole-brain analysis would allow for a broader exploration of neural associates of individual differences in memory behavior that would help to determine the degree to which the present findings are exclusive to pHPC, but as a trade-off, it would be more limited in its statistical power and also become more susceptible to the pitfalls of brain wise association studies that often lead to inflated effect sizes (Marek et al., 2022).

A related limitation is that the *a priori* pHPC region, combined with the voxel size of the acquired data, did not allow us to localize effects to specific hippocampal subfields, which have been shown to exhibit different effects in memory tasks (Miller et al., 2020; Stark et al., 2021). Future experiments, perhaps conducted at 7T and with sub-millimeter voxel sizes, would offer a more specific view of the basis for the observed individual differences.

We calculated task-induced activation levels against a resting state baseline. Although there is evidence that it constitutes an interpretable baseline that aligns with neural baseline measures and metabolic baseline as established by PET (Gusnard et al., 2001; Raichle & Gusnard, 2005), others have argued that its unconstrained nature poses difficulties because the hippocampus is always actively encoding and retrieving information (Martin et al., 1997; Stark & Squire, 2001; Stark et al., 2021). In contrast, foil task baselines constrain cognitive activity more overtly and, if multiple baseline tasks are employed, would provide an opportunity to isolate task components that are differentially correlated with pHPC activity across. Indeed, one previous study that used an active foil task as baseline also localized individual differences in CVLT-II performance to the hippocampus (Johnson et al., 2001), providing additional support for our conclusions.

### Conclusions

In sum, our findings converge with an earlier body of work showing a close connection between the hippocampus and individual differences in memory-related cognition. By identifying a replicable negative correlation between task-induced hippocampal activity and scores on a general memory evaluation, the present findings indicate the pHPC-verbal memory relationship as both consistent and reliable. By externally replicating Wig et al. (2008), the present findings now provide a motivation for future research into the nature of why task-induced pHPC changes reflect individual differences, particularly in the counter-intuitive direction that was observed, and how this might be used to better understand expression and risk of memory impairment.
